# Drug related problems in clinical practice: a cross-sectional study on their prevalence, risk factors and associated pharmaceutical interventions

**DOI:** 10.1038/s41598-020-80560-2

**Published:** 2021-01-13

**Authors:** Noe Garin, Nuria Sole, Beatriz Lucas, Laia Matas, Desiree Moras, Ana Rodrigo-Troyano, Laura Gras-Martin, Nuria Fonts

**Affiliations:** 1 Department of Pharmacy, Hospital de La Santa Creu i Sant Pau, Universitat Autònoma de Barcelona, Barcelona, Spain; 2 Centro de Investigación Biomédica en Red de Salud Mental (CIBERSAM), Instituto de Salud Carlos III, Madrid, Spain; 3 School of Health Science Blanquerna, Universitat Ramon Llull, Barcelona, Spain; 4 Department of Internal Medicine, Hospital de La Santa Creu i Sant Pau, Universitat Autònoma de Barcelona, Barcelona, Spain; 5 Department of Respiratory Medicine, Hospital de La Santa Creu i Sant Pau, Universitat Autònoma de Barcelona, Barcelona, Spain

**Keywords:** Diseases, Health care, Medical research, Risk factors

## Abstract

Drug-related problems (DRP) cause preventable negative health outcomes, especially during hospital admissions. The aim of our study was to examine the prevalence and characteristics of DRP in regular clinical pharmacy, as well as to determine those factors associated with a higher risk of DRP in the hospital setting. We analyzed data from a standardized registry database of regular pharmacy practice (2015- 2016). DRP were classified according to the Pharmaceutical Care Network Europe v6.2 classification. Cross-sectional data were obtained from 1602 adults admitted to medical wards. Crude and adjusted binary logistic regressions were performed to identify associations between potential risk factors and DRP. Overall DRP prevalence was high across medical specialties (45,1%), in a population characterized by advanced age, polypharmacy and multimorbidity. Problems leading to DRP were mainly classified into two domains (effectiveness and adverse reactions), being drug and dose selection the most frequent causes. Interventions were accepted and DRP were totally or partially solved in 74.1% and 4.81% of cases, respectively. In the adjusted model polypharmacy, allergies, BMI > 25 kg/m2 and clearance < 30 mL/min were associated with a higher risk of DRP. The participation of clinical pharmacists into multidisciplinary teams promotes the detection and solution of DRP. Polypharmacy, obesity, renal impairment and allergy are associated with a higher risk of DRP during admission.

## Introduction

Multimorbidity, the presence of several co-occurring conditions, is present in about 70% of the older adult population and becomes a major clinical and financial challenge for healthcare systems^1,2^. For example, most of the hospital medical admissions are the result of chronic diseases in the older adults^3,4^. There is a need of a comprehensive approach including the social sphere, nutrition and pharmacotherapy, to face with the increasing requirements of multimorbidity patients^5^.

Pharmacotherapy has been associated with negative health outcomes such as adverse effects, interactions, adherence problems, functional decline, cognitive problems, falls, urinary incontinence and metabolic or nutritional problems^6–13^. The risk of these problems increases with the number of drugs. Polypharmacy, defined as the use of more than four or five drugs, occurs in 40% of the adults over 65 years old^14–16^. Prevalence of polypharmacy reaches up to 90% of adults over 75 years at the moment of hospital admission^17^. Besides, during hospitalization, drug changes and new medicines for acute health problems will pose a higher risk of negative health outcomes. Up to 40% of hospitalized patients suffer from drug-related iatrogenesis^6^, emerging as the fourth to sixth mortality cause at this healthcare level^18^.

Several factors may significantly increase the risk of suffering a drug-related problem (DRP), defined as “an event or circumstance involving drug therapy that actually or potentially interferes with desired health outcomes”^19^ as previously described, for example, in experiences of care transitions across the continuum of care^20,21^. In the hospital setting, DRP may occur at all stages, from admission to discharge^20–25^. Certain conditions, drugs in specific therapeutic groups and variability of pharmacology knowledge across healthcare professionals could also be related to DRP^22,24,26–28^. However, there is controversy on the impact of these variables and others such as gender, age, social factors or readmissions on the risk of developing DRPs, especially in clinical practice^24,26,27,29–31^.

Fortunately, a substantial proportion of DRP can be prevented^32–34^. Pharmacy practice implies the review of prescriptions and relevant clinical data of hospitalized patients to optimize the effectiveness and safety of treatments. The incorporation of hospital pharmacists into multidisciplinary teams has been shown to increase the detection of DRPs according to research^35–40^. Interventions described in research studies focusing on DRP are varied and cover a broad range of aspects, such as medication reconciliation, medication adherence, dose adjustment or therapeutic indication^20,41–49^. However, activities in real clinical practice are neither homogeneous nor standardized and data collection, such as the prevalence or the characterization of DRPs, is unusual.

In particular, the study of DRP in patients admitted to medical wards results of great interest as these patients may be at a higher risk of DRP due to several factors: acute conditions leading to the admission, advanced age with high burden of chronic comorbidities, younger patients with severe diseases, polypharmacy, risk of renal impairment, frequent changes in drug treatment and length of the stay^29,37,50^. Studies focusing on medical units have historically tended to focus on specific medical fields or ambulatory patients^51–57^. Also, many studies on medical wards are research projects that may not reflect real-life practice as there may have some of the following limitations: prospective studies with restrictive inclusion criteria, specific protocols and teaching programmes, small sample sizes, use of automatized DRP alerts without direct pharmacist intervention, poor methodology description of the pharmaceutical care process, lack of validated registration tools and reliable information in retrospective analyses, lack of DRP risk factors analysis or study of a limited list of potential factors, short study duration or pathology/drug-centered rather than patient-oriented approach^26,27,38,40,44,45,47–49,58,59^. Also, only a few studies have explored the degree of acceptance of recommendations by the medical team^35–37,39,40^.

The detection and characterization of DRPs, the study of their causes and the evaluation of the associated interventions are of special interest in daily clinical practice, especially in the hospital medical wards due to the high risk of iatrogenesis. An adequate evaluation should consider validated DRP classification systems, representative samples and study periods long enough to draw valid conclusions. Thus, the current study aims to examine the prevalence and characterize DRPs in regular clinical pharmacy as well as to determine those factors associated with a higher risk of DRPs in the hospital setting.

## Methods

### Study design

This hospital-based, observational, cross-sectional study was conducted in a 700 bed University Hospital (March 2015-February 2016). This reference hospital provides care to a population of 405,000 people in Barcelona, Spain.

### Sample procedures

The sample included all adults over 18 years old admitted to the medical wards during the study period: Internal Medicine, Gastroenterology, Geriatrics, Neurology, Pneumology. Cardiology, Oncology and Haematology were excluded due to the presence of transplanted patients and special characteristics in relation to pharmacotherapy.

**Figure 1:**
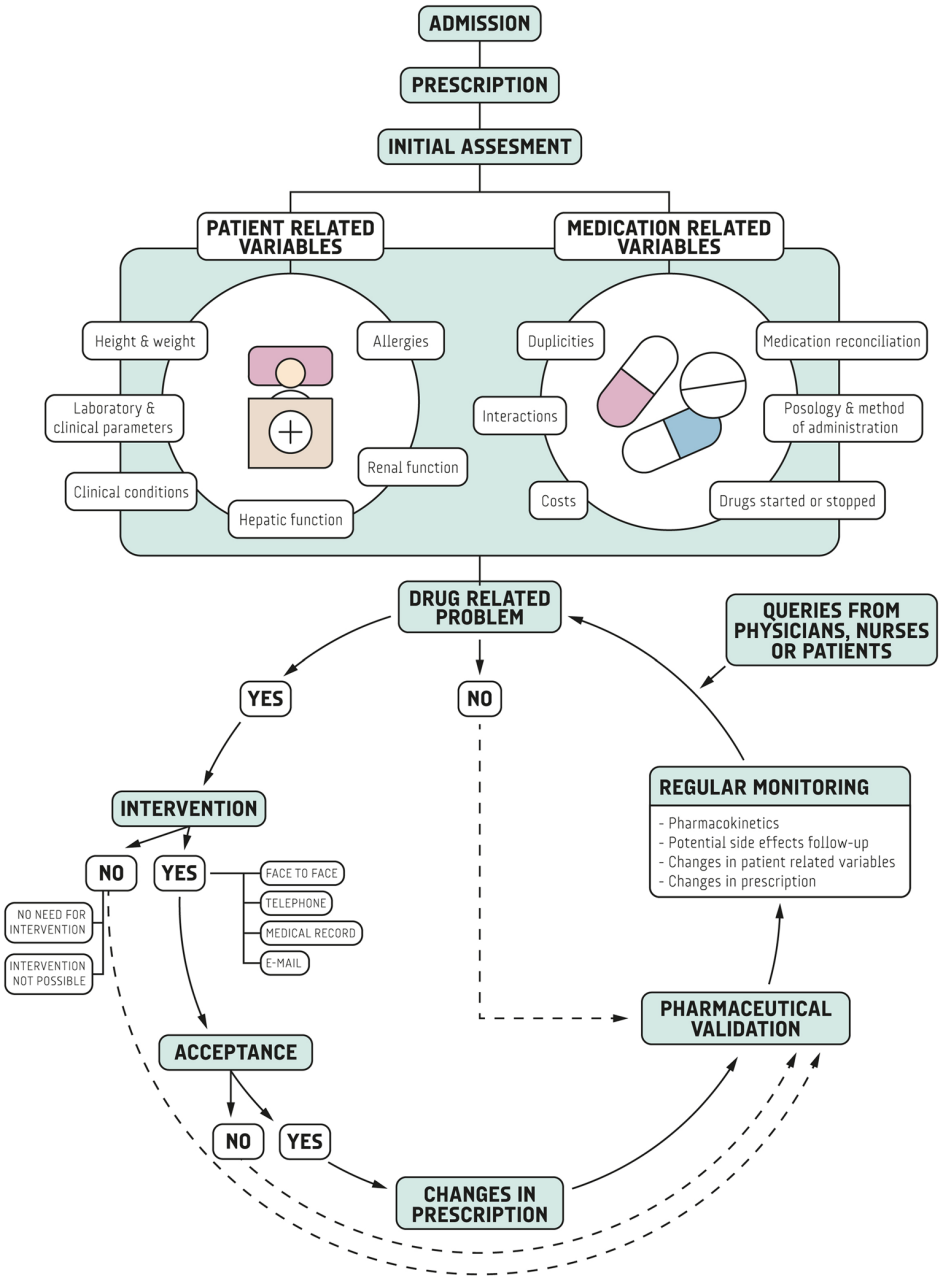
Pharmaceutical care workflow diagram.

The study consisted of the assessment of activities and interventions made and registered in regular clinical pharmacy practice. Briefly, three pharmacists performed regular pharmaceutical care activities following their standard workflow (Fig. 1). Firstly, they assessed specific aspects regarding pharmacotherapy at the time of admission and subsequent changes in prescription, including a wide range of activities (i.e. medication reconciliation, allergy check, indication, posology) (Appendix 1). Secondly, certain patients were selected for daily follow-up based on pre-specified criteria (i.e. pharmacokinetics monitoring, risk of adverse effects, potential interaction, renal impairment) or any other clinical criteria (Appendix 1). Additionally, pharmacists received queries raised by physicians, nurses, caregivers or patients. All these activities resulted in specific pharmaceutical interventions.

All patients assessed throughout the aforementioned steps were registered in the pharmacy work database, as part of their standard practice, including selected variables. Pharmaceutical interventions were also registered for those patients with detected DRP.

### Data collection and variable definitions

Data were obtained from a standardized registry database of regular pharmacy practice. Variables collected in the database were selected aiming to maximize pharmacotherapeutic utility of the data, taking into consideration clinical criteria and the reviews on factors related to DRP by Alomar and Kaufmann et al.^24,30^. Each of the variables described in the reviews was evaluated and those not applicable to the practice context were discarded (i.e. visual impairment, non-adherence; Appendix 2). Additional clinical variables of interest were included by consensus (Appendix 2).

Sociodemographic information included gender, age, country of birth, weight, height, body mass index (BMI). General clinical variables comprised creatinine clearance (Cockcroft-Gault), liver failure, number of chronic conditions, Charlson comorbidity index, allergies and medical specialty. Variables related to the use of health services were the number of last 12-month hospital admissions and coming from a nursing home. Pharmacotherapeutic variables were polypharmacy at admission (number of drugs), lack of information on regular treatment, monitoring drugs (amikacin, carbamazepine, cyclosporine, digoxin, everolimus, gentamicin, lithium, phenytoin, phenobarbital, sirolimus, tacrolimus, valproate, vancomycin), DRP detection, DRP description according to the Pharmaceutical Care Network Europe (PCNE) v6.2 DRP classification^19^, medication reconciliation problem, ATC group and an open field to be used in case further clarification was needed.

### Statistical analyses

Due to the retrospective nature of the study, a priori sample size estimation was not possible. However, after data collection was finished, we calculated the theoretical needed sample size with the GRANMO calculator to confirm the adequacy of our ulterior analysis. According to the bibliography, DRP in hospitalizations range from 20 to 80%^26–60,62^. A minimum sample size over 385 participants was needed considering an infinite population, 5% precision, 95% CI and a DRP prevalence of 50%. Hence, the sample obtained in our study was considered adequate for the analysis.

Frequencies, proportions, range, mean, SD, CIs and cross tabulations were applied for descriptive analysis. χ2 Test, Fisher's exact test and t-test were used to measure differences in prevalence of sociodemographic and clinical variables across DRP presence. T test, one-way ANOVA and simple linear regression were used to assess differences in polypharmacy across clinical and socio-demographic variables (age, gender, country, BMI, baseline chronic conditions, allergies, last-12 month admissions, creatinine clearance, liver failure, coming from nursing home, estimated 10-year survival, medical department and missing information defined as lack of data regarding chronic treatment in clinical records). Crude and adjusted binary logistic regressions were used to examine the relationship between theoretical factors related with DRP and their presence in our sample. The multivariate logistic regression model included those variables with an association in the bivariate analyses defined as *p* < 0.1. We fitted additional regression models for combinations of variables to test whether potential theoretical interactions were present with regard to the dependent variable: presence of DRP. Multicollinearity was also tested, defined as a condition index > 20 or VIF > 10. Both the presence of interactions (except number of chronic conditions with renal function) and multicollinearity were rejected. Results are reported as unadjusted and adjusted ORs with 95% CI. We conducted additional sensitivity analyses by age group (< 60 years, 60–69 years, ≥ 70 years). There were no missing data for most of the variables. Information on BMI and renal function were missing in 0.99% and 0.56% of the participants. These rates were considered low. We did not impute these variables as we could not guarantee whether these data were missing at random. Analyses were performed with IBM SPSS statistics V.19.

## Results

### Participant characteristics

Overall, data from 1602 hospital admissions were reviewed and registered. Participants mean age was 72.8 years (SD:15.09). Most individuals were Spanish, 44.8% of them being women. Mean number of baseline chronic conditions was 5.99 (SD:3.06), BMI was greater than 25 kg/m2 in 17.5% of cases, and renal function was below 30 mL/min in 15.5% of the sample. As for additional healthcare data of interest, 8.8% of the admissions came from a nursing home, 44.4% have had a previous admission over the last 12 months and 2.9% were reported as having missing information on previous treatments. A summary of the full list of sociodemographic and clinical data is available at Table 1.

**Table 1:** Description of the sample.

Variables	Categories	Patients without DRP	Patients with DRP	Total sample	*p* value
Gender (n, %)	Female	408 (46.4)	310 (42.9)	718 (44.8)	0.170
	Male	472 (53.6)	412 (57.1)	884 (55.2)	
Age (years; mean, SD)		72.67 (15.20)	72.92 (14.97)	72.78 (15.09)	0.747
Country (n, %)	Spain	838 (95.2)	694 (96.1)	1532 (95.6)	0.383
	Other	42 (4.8)	28 (3.9)	70 (4.4)	
Allergy (n, %)	Yes	176 (20.0)	180 (24.9)	356 (22.2)	0.018
	No	704 (80.0)	542 (75.1)	1246 (77.8)	
BMI (n, %)	< 18	212 (24.5)	155 (21.6)	367 (23.1)	0.003
	18–25	529 (61.0)	413 (57.4)	942 (59.4)	
	≥ 25	126 (14.5)	151 (21.0)	277 (17.5)	
Polypharmacy (mean, SD)		7.65 (4.65)	8.77 (4.71)	8.15 (4.71)	0.000
Missing information (n, %)	Yes	25 (2.8)	22 (3.0)	47 (2.9)	0.808
	No	855 (97.2)	700 (97.0)	1555 (97.1)	
Hepatic impairment (n, %)	Yes	95 (10.8)	83 (11.5)	178 (11.1)	0.306
	No	785 (89.2)	635 (88.5)	1424 (88.9)	
Renal function (n, %)	< 30	124 (14.2)	123 (17.1)	247 (15.5)	0.148
	30–60	282 (32.3)	242 (33.7)	524 (32.9)	
	≥ 60	468 (53.5)	354 (49.2)	822 (51.6)	
NCC (mean, SD)		5.76 (3.01)	6.27 (3.09)	5.99 (3.06)	0.001
Coming from NH (n, %)	Yes	76 (8.6)	65 (9.0)	141 (8.8)	0.802
	No	803 (91.4)	657 (91.0)	1460 (91.2)	
Last 12 months HA (n, %)	Yes	365 (41.5)	347 (48.1)	712 (44.4)	0.008
	No	515 (58.5)	375 (51.9)	890 (55.6)	
Department (n, %)	Digestive	80 (9.1)	72 (10.0)	152 (9.5)	0.828
	Geriatrics	195 (22.2)	166 (23.0)	361 (22.5)	
	Internal Medicine	253 (28.8)	198 (27.4)	451 (28.2)	
	Neurology	181 (20.6)	137 (19.0)	318 (19.9)	
	Respiratory	171 (19.4)	149 (20.6)	320 (20.0)	
Estimated 10-year survival (Charlson Index; mean, SD)		27.37 (34.69)	23.32 (33.36)	25.54 (34.15)	0.018

Frequencies, proportions, means and SDs are displayed. χ^2^ Test (for 2 × N tables) and t-test (for continuous variables) were performed to compare across the presence of DRP. Missing information: lack of data regarding chronic treatment in clinical records. *BMI* body mass index, *CI* confidence interval, *DRP* drug related problem, *HA* hospital admissions, *NCC* number of chronic conditions, *NH* nursing home, *SD* standard deviation.

**Figure 2:**
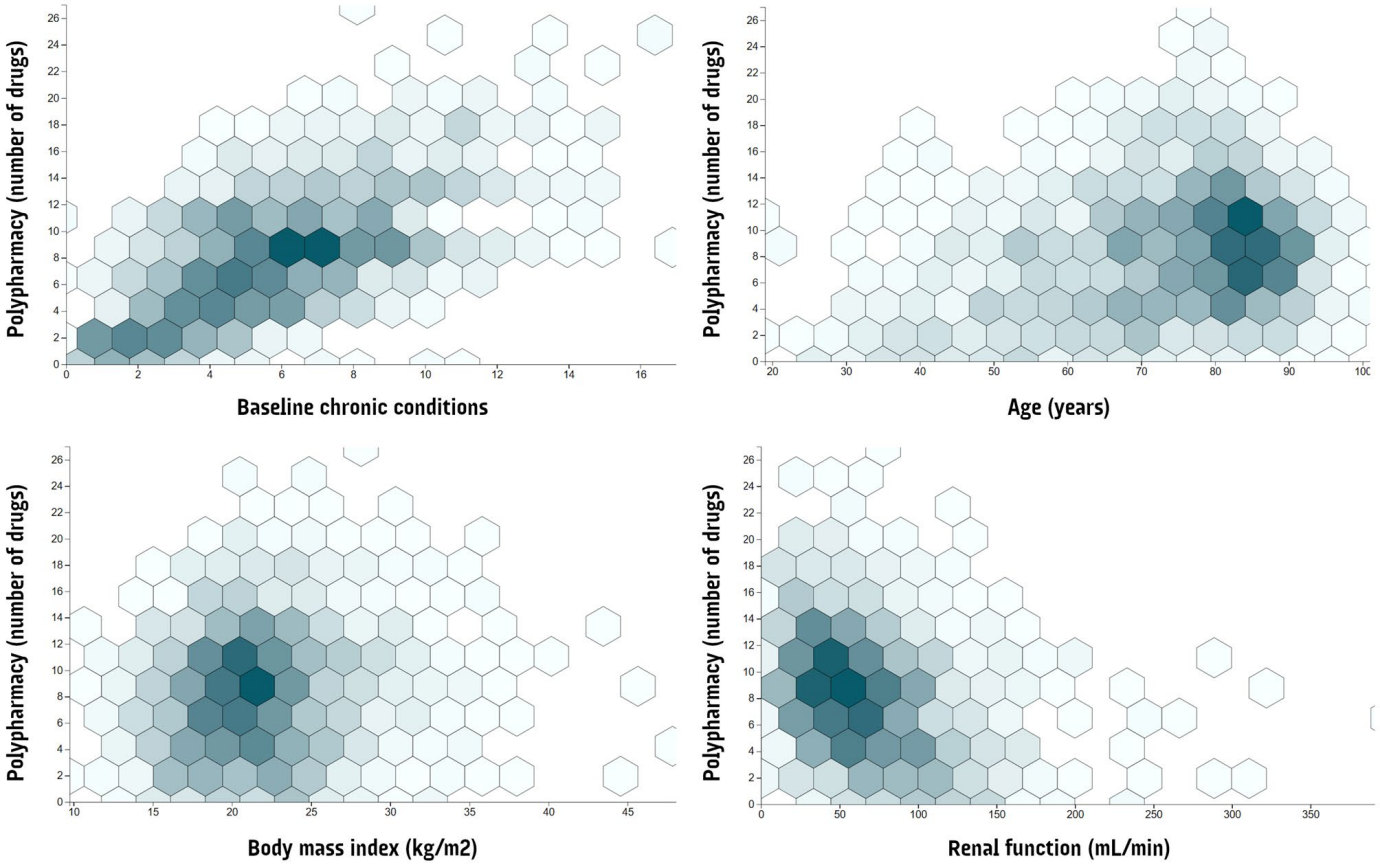
Hexagonal binning chart of distribution of Polypharmacy across selected variables. The graph visually cluster the most populated areas on a scatterplot (colour intensity increases with frequency).

Regarding polypharmacy, mean number of drugs was 8.15 (SD:4.71). Certain variables showed a positive association with the degree of polypharmacy: last-12 months admissions (*p* = 0.001), coming from nursing home (*p* = 0.007), estimated 10-year survival below the median (*p* = 0.001), older age (*p* = 0.001), baseline chronic conditions (*p* = 0.001), and decreased renal function (0.001). Differences in polypharmacy were also found across departments (Respiratory = Geriatrics > Internal Medicine > Digestive = Neurology; *p* = 0.001); and across BMI (overweight > normoweight > infraweight; *p* = 0.001). For the specific distribution of polypharmacy across selected quantitative variables see Fig. 2.

### Description of DRP

DRP were detected in 722 (45.1%) patients and their prevalence differed across BMI, allergy, polypharmacy, multimorbidity, last 12 months admissions and the estimated 10-year survival in (Table 1). DRP were related to medication reconciliation in 38.4% of the cases.

According to the PCNE V6.2 classification, most of the problems leading to DRP were included into two domains: effectiveness and adverse reactions. The principal problem was “toxic adverse drug-event”, followed by “untreated indication” and “effect of treatment not optimal” (Table 2). Regarding the cause of DRP, the most frequent domains were “drug selection” and “dose selection”. The leading causes were “indication for drug-treatment not noticed”, “pharmacokinetic problem requiring dose adjustment” and “drug dose too high”.

**Table 2:** Description of the drug related problems, associated pharmaceutical interventions and outcomes.

Problems		Causes		Interventions at the prescriber level		Interventions at the drug level		Outcome of intervention	
Category	n (%)	Category	n (%)	Category	n (%)	Category	n (%)	Category	n (%)
Toxic drug event	292 (24.64)	Indication for drug-treatment not noticed	255 (21.52)	Intervention proposed, approved by Prescriber	788 (66.50)	Dosage changed	434 (36.62)	Problem totally solved	878 (74.09)
Untreated indication	273 (23.04)	Pharmacokinetic problem requiring dose adjustment	205 (17.30)	Intervention proposed, outcome unknown	165 (13.92)	No intervention (no change)	356 (30.04)	Outcome intervention not known	186 (15.70)
Effect of drug treatment not optimal	217 (18.31)	Drug dose too high	158 (13.33)	Prescriber informed only	69 (5.82)	New drug started	147 (12.41)	Problem partially solved	57 (4.81)
Adverse drug event (non-allergic)	144 (12.15)	Drug dose too low	102 (8.61)	Intervention proposed, not approved by Prescriber	69 (5.82)	Drug stopped	109 (9.20)	No need or possibility to solve the problem	25 (2.11)
No effect of drug treatment/therapy failure	80 (6.80)	Inappropriate drug (incl. contra-indicated)	66 (5.57)	No Intervention	53 (4.47)	Drug changed	62 (5.23)	Problem not solved, lack of cooperation of prescriber	23 (1.94)

Only the five categories most frequently found are listed. Total number of DRP: 1185.

We divided interventions results into prescriber and drug levels. As for the first one, intervention was proposed and approved in two thirds of the cases, while not approved in only 5.8% of cases. Outcome was unknown in 13.9% interventions (for more categories, see Table 2). Pharmaceutical intervention results covered a wide range of aspects, such as: drug dose adjustment in patients with renal or hepatic impairment (e.g.: levofloxacin, simvastatin, paroxetine), treatment changes due to drug-drug interactions (e.g.: valproate with carbapenem antibiotics; high doses of simvastatin with diltiazem), need of dose adjustments (e.g.: low doses of antimicrobials for CNS infections infections in the central nervous system, excessive drug dose leading to potential harms), untreated indications (e.g.: hyperglycaemia, high-blood pressure, ACE Angiotensin-converting enzyme inhibitor induced hyperkalemia), contraindications (e.g.: oral bisphosphonates in patients with dysphagia), medication reconciliation (e.g.: dose/frequency, drugs to be stopped), IV intravenous administration issues (e.g.: excessive infusion rate of vancomycin or electrolytes; wrong serum for dilution, Y-Y incompatibilities), oral administration issues (e.g.: recommendation of available presentations for dysphagia, medicines to be taken on an empty stomach such as alendronic acid), contraindication or excessive duration treatment (e.g.: excessive antibiotic drug length, antihypertensive therapy in patients with low blood pressure), pharmacokinetics monitoring (e.g.: dosage increase/decrease or discontinuation of vancomycin, gentamicin, amikacin, etc.), wrong drug prescribed (e.g.: methimazole for metamizole), cost-efficacy interventions (e.g.: changes of low molecular weight heparins according to the hospital formulary). At a drug level, dosage was changed in 36.6% of cases, a new drug was started in 12.4% and the drug was stopped in 9.2% of the interventions, while 30.04% resulted in no changes. As for the final health outcomes, DRP were considered to be totally solved in 74.1% and partially solved in 4.8% of cases. The outcome was not known in 15.7% of interventions.

**Figure 3:**
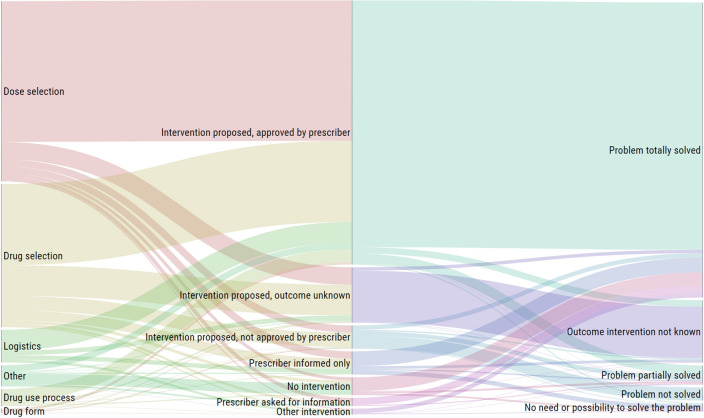
Interventions on DRP pathway. Alluvial diagram representing the flow and correlation throughout the dimensions “cause, intervention at prescriber level, and outcome” of DRPs. Height size is proportional to the number of DRP in each dimension.

Considering pharmacist interventions on DRP as a continuum and clustering categories according to the domains of the PCNE V6.2, certain patterns were observed in the DRP pathway “cause → intervention → outcome” (Fig. 3).

Also, the five most frequent Anatomical Therapeutic Chemical (ATC) Classification System groups in the detected DRP (n = 1185) were: J “Antiinfectives for systemic use” (n = 337; e.g.: vancomycin, levofloxacin, amikacin), N “Nervous system” (n = 206; e.g.: valpropate, citalopram, sertraline), C “Cardiovascular system” (n = 195; e.g.: digoxin, simvastatin, enalapril), B “Blood and blood forming organs” (n = 114; e.g.: acetylsalicylic acid, enoxaparin, bemiparin) and A “Alimentary tract and metabolism” (n = 99; e.g.: omeprazole, potassium chloride, vitamin D). (Appendix 3).

### Factors associated with DRP

Table 3 shows the crude and adjusted logistic regression odds ratios for the association between potential factors leading to DRP and the actual presence of these problems (n = 1602). In the crude analysis, number of diseases, previous last 12 month admissions, polypharmacy, allergies and BMI > 25 kg/m^2^ were associated with a great prevalence of DRP. A higher estimated 10-year survival was associated with a lower risk. In the adjusted model only polypharmacy, allergies, BMI > 25 kg/m^2^, renal function < 30 mL/min and the interaction “multimorbidity*renal function < 30 mL/min” were still associated with a higher risk of DRP (OR 1.040 [CI 1.010–1.071]; OR 1.281 [CI 1.004–1.634); OR 1.515 [CI 1.143–2.007]; OR 2.146 [1.200–3.837]; OR 0.908 [CI 0.838–0.985]) respectively).

**Table 3:** Correlates of drug-related problems estimated by multivariable logistic regression.

Variable	Categories	OR crude (95% CI)	*p*	AOR (95% CI)	*p*
Department	Respiratory	1.000	–	–	–
	Digestology	1.003 (0.702 to 1.521)	0.870	–	–
	Geriatrics	0.977 (0.722 to 1.321)	0.880	–	–
	Internal Medicine	0.898 (0.674 to 1.198)	0.465	–	–
	Neurology	0.869 (0.636 to 1.187)	0.377	–	–
Age		1.001 (0.995 to 1.008)	0.747	0.993 (0.983 to 1.002)	0.132
Gender	Male	1.000	–	1.000	–
	Female	0.870 (0.714 to 1.061)	0.170	0.861 (0.689 to 1.002)	0.164
Last-12 months HA	No	1.000	–	1.000	–
	Yes	1.306 (1.071 to 1.592)	0.008	1.110 (0.897 to 1.373)	0.336
Country	Spain	1.000	–	–	–
	Foreigner	0.805 (0.494 to 1.312)	0.384	–	–
Coming from NH	No	1.000	–	–	–
	Yes	1.045 (0.739 to 1.479)	0.802	–	–
Missing information	No	1.000	–	–	–
	Yes	1.075 (0.601 to 1.923)	0.808	–	–
Hepatic impairment	No	1.000	–	–	–
	Yes	1.073 (0.785 to 1.467)	0.657	–	–
Estimated 10-year survival (Charlson Index)	≤ Median	1.000	–	1.000	–
	> Median	0.808 (0.643 to 1.016)	0.069	0.933 (0.670 to 1.300)	0.684
Polypharmacy		1.052 (1.030 to 1.075)	0.000	1.040 (1.010 to 1.071)	0.009
Allergies	No	1.000	–	1.000	–
	Yes	1.328 (1.049 to 1.682)	0.018	1.281 (1.004 to 1.634)	0.047
BMI	18–25	1.000	–	1.000	–
	< 18	0.936 (0.0734 to 1.195)	0.598	0.949 (0.731 to 1.232)	0.695
	> 25	1.535 (1.173 to 2.009)	0.002	1.515 (1.143 to 2.007)	0.004
NCC		1.088 (1.037 to 1.1142)	0.001	1.037 (0.975 to 1.103)	0.242
Renal function	≥ 60	1.000	–	1.000	–
	30–60	1.406 (0.650 to 3.042)	0.386	1.742 (0.756 to 4.015)	0.192
	< 30	1.706 (1.013 to 2.874)	0.045	2.146 (1.200 to 3.837	0.010
Interactions					
NCC*renal function	NCC* ≥ 60	1.000	–	1.000	–
	NCC*30–60	0.963 (0.871 to 1.065)	0.468	0.954 (0.858 to 1.061)	0.384
	NCC* < 30	0.922 (0.854 to 0.996)	0.039	0.908 (0.838 to 0.985)	0.021

Results refer to univariate and multivariable logistic regression for the total sample. Age and polypharmacy were included as continuous variables. *AOR* adjusted odds ratio, *BMI* body mass index, *CI* confidence interval, *HA* Hospital admissions, *NCC* number of chronic conditions, *NH* nursing home, *OR* odds ratio.

Sensitivity analyses by age group showed similar results to those of the global analyses. For only a few variables, results maintained directionality and tendency but did not reach the statistical significance: Group 18–59 years: renal function < 30 mL/min [OR 3.365 (0.378–29.971); *p* = 0,2]; Group 60–69 years: BMI > 25 kg/m^2^ [OR 1.685 (0.902–3.148);*p* = 0,1]; polypharmacy [OR 1.202 (0.945–1.102); *p* = 0,6], Group 70 + years: allergy [OR 1.183 (0.884–1.585); *p* = 0,2]. Sample size of the youngest groups was smaller than the oldest group (< 60 years: 300 patients; 60–69 years: 246 patients; 70 + years: 1056 patients).

## Discussion

To the best of our knowledge, this is one of the few studies to evaluate DRP prevalence and risk factors in real clinical practice during hospital admission in the context of a standardized pharmaceutical care programme, including a validated register of DRP interventions with a global representation of medical specialties. We found a high prevalence of DRP, most being caused by drug or dose selection, in a sample that highlights the worldwide demographical trends of ageing along with multimorbidity and polypharmacy. Pharmacist interventions were accepted in most cases, preventing potential negative health outcomes. As for potential risk factors, only polypharmacy, renal impairment, allergies and high BMI were associated with a higher prevalence of DRP. The main strengths of our study are the large sample size, the standardized procedures of clinical pharmacists throughout the admission in regular clinical pharmacy, the use of a validated DRP classification, the assessment of a comprehensive list of potential DRP risk factors and the inclusion of the most relevant medical specialties.

Our study found a great proportion of admissions with DRPs, a 45.1% of the cases. Most of the evidence available at a hospital level have focused on specific medical fields^51–53^, being limited to ambulatory patients in numerous occasions^54–56^. There are, however, some studies focusing on medical specialties, which found a DRP prevalence ranging from 15 to 81%^26,27,60–62^. Our results fit well into the related literature although it should be noted that literature on geriatrics show the higher results^29,58,63^. In contrast, results from studies which use automatized DRP alerts in computerized prescriber order entry systems have shown lower prevalence in previous studies^26,40^. Thus, these systems should be considered as tools to complement pharmacy practice rather than a substitution of clinical pharmacist functions due to the complexity of hospitalized patients.

Our study highlights the complexity of patients admitted in medical wards, with an average age over 72 years, in line with recent literature^26,27,60–62^. The World Health Organization has recognized demographic transitions as a major priority due to its burden at a health, social and economic levels^64^. Ageing involves a complex set of complications reflected in our results, such as multimorbidity, polypharmacy or functional impairment. Mean number of chronic conditions in our study was six and ranged up to 16 per patient, intimately related to a low expected 1-year survival of 25%. Most of the previous literature focusing on regular clinical pharmacy practice care in medical wards reported minimal data at this level as they had a more drug-centred approach^26,27,60–62^. Polypharmacy stands out as one of the most valuable variables of our study, ranging up to 26 drugs per patient, due to their potential harmful effects. In a context with a high burden of polypharmacy, there is need to move from the classical thresholds of 4+ or 5+ drugs^14–16^ to a more realistic linear approach, which we used in our analyses. A further complex approach, considering polypharmacy as a qualitative aspect is still being discussed. In the end, these characteristics and those regarding the own health system, such as communication across healthcare levels, will define the objectives for a specific patient and the type of interventions in drug treatment.

Regarding pharmacists’ interventions, acceptance accounted for almost 70% cases. This issue has not been properly assessed in many previous studies on DRPs in regular clinical practice^26,27,60,61^. Those studies documenting this data show similar interventions acceptance in regular clinical practice^62^ or even higher figures in prospective research studies compared with our results^58^. Also, final outcomes of the interventions showed total or partial resolution of the DRPs in our study, reinforcing the value of our results. Interventions registers are essential, as in other healthcare areas, to document professional activity and to assess the suitability of the approach being taken to eventually improve healthcare outcomes^65^. Despite its possible benefits, registries of clinical pharmacy have normally been present in specific areas, such as those linked to a computerized DRP alert system or nationwide voluntary reporting systems^40,66^.

Finally, one of the most relevant issues regarding DRPs is understanding their potential underlying factors to optimize interventions and preventive measures. As previously stated, there are many possible factors suggested by the literature. Certain theoretical frameworks or reviews have developed lists of risk factors but they may not totally apply to the care of admitted patients^24,30^. For example, issues such as self-medication, visual impairment, civil status or educational level would not be relevant from the acute drug management perspective. In contrast, we found that only polypharmacy, renal impairment, allergies and high BMI were associated with DRPs. A review of the literature on this topic at a hospital level used definitions not totally comparable to ours, i.e. medication errors, but polypharmacy and renal function also resulted to be risk factors^29^. We hypothesize that the idiosyncrasy and complexity of inpatients in our or similar environments may diminish the impact of alternative factors.

Our study has limitations. First, its cross-sectional nature identifies associations but does not allow causal relationships to be determined. Second, our results may differ from geographical areas with different healthcare characteristics, especially in terms of pharmaceutical care implementation. Nor can they be extrapolated to other specialties or primary care. Third, this study refers to DRPs, which by definition have a potential nature. Interventions where made in all detected DRPs, so that it is not possible to quantify the real negative health outcomes. As regular practice, it is not considered ethical to stop performing clinical activities for this reason. Fourth, administration errors, if not notified by nursing staff or detected by the pharmacist staff, were not registered. This limitation has been described in other studies previously. Fifth, underdetection may have happened to some extent, especially for infrequent or unfamiliar DRPs. This problem is partly covered by using a complete, standardized and validated classification system. Another factor that can influence underreporting is professional experience. Also, we did not include potential factors leading to DRP which were considered not applicable to practice context (e.g.: education level, visual impairment). These are relevant factors in primary care and could potentially impact on the results of specific hospitalizations. Moreover, our analyses have shown similar tendencies in the global analysis compared with the sensitivity age group analyses. However, some results did not achieve statistical significance in the younger groups, probably because sample sizes were smaller. Further research should focus on possible differences across age groups. Also, verbal interventions have been associated with higher acceptance rates compared with written interventions. In our study, most of them were verbal but this variable was not recorded and its impact could not be analyzed. Finally, the clinical relevance of interventions was not assessed as it is not collected routinely in regular clinical practice but future studies should address this issue.

DRPs have become a major challenge for health care systems due to their clinical and economic impact, especially in the context of the current demographical ageing trends that imply a high burden of multimorbidity and polypharmacy. The prevalence of DRP in hospitalized patients admitted to medical wards is high regardless of the specialty, which highlights the need of providing pharmaceutical care to prevent negative health outcomes during hospitalization. Problems leading to DRPs are mainly related to effectiveness and adverse reactions, while the most frequent causes are drug and dose selection. Thus, these domains should be prioritized in both pharmaceutical care programmes and general educational activities. The participation of clinical pharmacists into the multidisciplinary team promotes the detection and solution of DRP in the majority of cases, and should be considered as a rule in general clinical practice. Finally, only a limited number of factors may be associated with a higher risk of developing DRPs in the hospital setting, such as polypharmacy, allergies, BMI > 25 kg/m2 and renal function < 30 mL/min, which could be useful to prioritize actions. Better understanding of these issues may facilitate the implementation of general approaches in diverse settings and the study of these interactions in the future.

### Ethics statement

The study was approved by the Clinical Research Ethics Committee of the Hospital de la Santa Creu i Sant Pau, Barcelona, Spain. This Ethics Committee waived the need to obtain informed consent due to the retrospective nature of the data, coming from a standardized regular practice database, and the anonymized analysis. All investigators worked according to the principles expressed in the Declaration of Helsinki.

## Supplementary Information

Supplementary Information 1.

Supplementary Information 2.

Supplementary Information 3.

## Data Availability

The datasets generated during and/or analyzed during the current study are available from the corresponding author on request.
