# Supplementary material for: Drug related problems in clinical practice: a cross-sectional study on their prevalence, risk factors and associated pharmaceutical interventions
Source: Sci Rep. 2021 Jan 13;11:883. doi: 10.1038/s41598-020-80560-2 (PMC7807048; doi:10.1038/s41598-020-80560-2)
Supplement: Supplementary file 1 — Supplementary Information 1. [file 41598_2020_80560_MOESM1_ESM.docx]

**Appendix 1. Standard workflow and description of pharmaceutical activities (including initial assessment at admission time and regular monitoring)**

| **Workflow phase** | **Activities** | **Characteristics** |
| --- | --- | --- |
| Initial assessment  Initial assessment (continuation)  Initial assessment (continuation) | Evaluation of patient related variables  Evaluation of patient related variables (continuation) | Anthropometric data:   - Weight - Height - Body Mass Index |
|  |  | Allergy and intolerance to drugs |
|  |  | Renal function:   - Serum creatinine, urea - Creatinine Clearance (Cockcroft-Gault Equation) - Serum electrolytes - Other: volume and aspect of diuresis, etc. |
|  |  | Hepatic function:   - Bilirubin - Transaminases - Alkaline phosphatase - Gamma glutamyl transpeptidase - Albumin - Other |
|  |  | Other laboratory, clinical and microbiological parameters:   - Temperature - Heart rate - Blood pressure - O^2^ saturation - Glycaemia - Blood analysis (including blood count, biochemistry test, coagulation test, serology, anaemia test, venereal diseases test, etc.) - Microbiology tests |
|  |  | Clinical conditions:   - Cause of admission - Chronic conditions - Patient’s health status   - Cognitive impairment   - Disability   - Prognosis   - Pain   - Digestive disorders   - Dysphagia   - Level of consciousness |
|  |  | Additional data of interest:   - Nasogastric tube - Gastrostomy - Jejunostomy - Urinary catheter - Other |
|  |  | Comorbidity and prognosis   - Charlson index - Physician’s comments on electronic records |
|  |  | Coming from nursing home (imply additional information beyond primary care records, usually obtained by the physician) |
|  | Evaluation of prescription and drug-related variables  Evaluation of prescription and drug-related variables (continuation) | Medication reconciliation:   - Chronic treatment was obtained from electronic prescription or by asking the patient, a family member or a caregiver. |
|  |  | Posology and method of administration:   - Dose - Frequency - Administration schedule (i.e.: at night, with meals, etc.) - Administration route and special related issues - Reconstitution and dilution fluid - Final concentration - Infusion rate - Other |
|  |  | Duplicities:   - Same active ingredient or different active ingredients from the same pharmacological family or from different families whose effects overlap leading to toxicity risk without clinical benefit. |
|  |  | Interactions:   - Drug-drug interactions - Drug-food interactions |
|  |  | Contraindications |
|  |  | Changes in treatment:   - Drugs started, stopped or modified at the moment of the admissions (assessment of indication) |
|  |  | Costs:   - Availability of a more cost-effective treatment |
| Regular monitoring during the admission  Regular monitoring during the admission  (continuation)  Regular monitoring during the admission  (continuation) | Evaluation of the changes in prescription  Evaluation of the changes in prescription (continuation) | Changes in prescription and validation:   - New drug started   - Indication   - Posology and method of administration     - Dose     - Frequency     - Administration schedule (i.e.: at night, with meals, etc.)     - Administration route and special related issues     - Reconstitution and dilution fluid     - Final concentration     - Infusion rate     - Other   - Duplicities     - Same active ingredient or different active ingredients from the same pharmacological family or from different families whose effects overlap leading to toxicity risk without clinical benefit.   - Interactions     - Drug-drug interactions     - Drug-food interactions   - Dose adjustment by renal function, hepatic function, or other clinical requirements   - Contraindications   - Costs     - Availability of a more cost-effective treatment   - Other - Drug stopped - Changes in:   - Dosage   - Frequency   - Timing of administration   - Route of administration   - Other |
|  | Monitoring of patient-related situations | Issues that may lead to drug-related problems or may require changes in prescription:   - Specific clinical conditions - Renal function - Abnormalities in electrolytes (i.e. hypokalaemia) - Other |
|  | Potential side-effects follow-up | Potential side-effects due to:   - The inherent toxicity of the drug (i.e. Linezolid-induced thrombocytopenia) - Interactions with other drugs - Interactions with food - Interventions not accepted with a clinical justification but potential risk of drug problems. |
|  | Pharmacokinetics  Pharmacokinetics  (continuation) | Therapeutic drug monitoring and recommendations for dose adjustments of the following drugs:   - Amikacin - Carbamazepine - Cyclosporine - Digoxin - Gentamicin - Lithium - Phenobarbital - Phenytoin - Sirolimus - Tacrolimus - Tobramycin - Valproic acid - Vancomycin. - Other: in particular cases assessment of other drugs. |
| Transversal or supplementary activities | Queries to the pharmacist | Phone calls, emails and direct queries made by:   - Physicians - Nurses - Other social or healthcare professional - Patient or caregiver |
|  | Social context issues | Intended to prevent problems in patients who need special interventions to ensure the access to medication and its correct use. |
