# Supplementary material for: Drug related problems in clinical practice: a cross-sectional study on their prevalence, risk factors and associated pharmaceutical interventions
Source: Sci Rep. 2021 Jan 13;11:883. doi: 10.1038/s41598-020-80560-2 (PMC7807048; doi:10.1038/s41598-020-80560-2)
Supplement: Supplementary file 2 — Supplementary Information 2. [file 41598_2020_80560_MOESM2_ESM.docx]

**Appendix 2. Selection of variables associated with DRPs in our study according to the literature and additional variables agreed by the authors**

| **Source** | **Section** | **Variable** | **Comments** |
| --- | --- | --- | --- |
| Alomar JM.  2012 (1) | Patient related factors | Age | Included |
|  |  | Gender | Included |
|  |  | Maternity status | Not applicable |
|  |  | Fetal development | Not applicable |
|  |  | Creatinine clearance | Cockcroft-Gault formula (mL/min) |
|  |  | Allergy | Included (yes/no) |
|  |  | Body weight and fat distribution | BMI (kg/m^2^) |
|  | Social related factors | Alcohol drinking | Not applicable (this variable is intended to assess interactions and other ADR related to alcohol consumption. Patients are not allowed to consume alcohol during the hospital admission so this variable would not be relevant) |
|  |  | Race and ethnicity factors | Country |
|  |  | Smoking | Not applicable (this variable is intended to assess interactions and other ADR related to smoking. Patients are not allowed to smoke during the admission so this variable would not be relevant. Cutaneous vasoconstriction by nicotine may decrease the rate of SC insulin absorption). |
|  | Drug related factors | Polypharmacy | Number of drugs at home (if not available, number of drugs at admission in the ward). |
|  |  | Drug dose and frequency | Not applicable (we considered this is a drug-related problem *per se*, which is one of the measures in our study. This would be, in turn, leading to ADR). |
|  | Disease related factors | Multiple diseases | Number of chronic conditions at admission |
| Kaufmann CP *et al*. 2015 (2) | Patient related factors | Experience of ADR | Not applicable |
|  |  | Medication that is difficult to handle | Not applicable |
|  |  | Self-medication | Not applicable |
|  |  | Impaired manual skills | Not applicable |
|  |  | Visual impairment | Not applicable |
|  |  | Non-adherence | Not applicable |
|  |  | Language issues | Not applicable |
|  | Drug related factors | Missing information | Information on regular drug treatment not available in electronic records or admission notes. |
|  |  | Polypharmacy | - |
|  |  | Medication that is difficult to handle | Not applicable |
|  |  | Selected drug groups | Drug and ATC (not analyzed in the study) |
|  |  | Medication with a narrow therapeutic window | Pharmacokinetic drug related |
|  | Disease related factors | Polymorbidity | Number of chronic conditions at admission |
|  |  | Renal impairment (<30 mL/min) | Cockcroft-Gault formula (mL/min) |
|  |  | Hepatic impairment | Included (yes/no) |
|  |  | Dementia | Not applicable. (Included as a condition in the multimorbidity variable). |
|  | Healthcare related factors | Missing information, half-knowledge of the patient, understanding of the patient | Not applicable |
| Additional variables considered by the authors | Patient related factors | Autonomy/disability | Not applicable |
|  |  | Cognitive status | Not applicable |
|  | Social related factors | Civil status | Not applicable |
|  |  | Education level | Not applicable |
|  | Drug related factors | Potential inappropriate medication | Not included as a variable. However, pharmacists assessed regularly medications considering the potential inappropriateness of drugs and relevant criteria during their practice. |
|  |  | Medication with a narrow therapeutic range during the admission | Included. All drugs with a narrow therapeutic range were monitored and included in the analyses if a DRP was present. However, the authors did not perform additional analyses for this specific variable. |
|  | Disease related factors | Charlson index (3-5) | Included as a measure of comorbidity and prognosis (ten year mortality) |
|  | Healthcare related factors | Coming from nursing home | Included (yes/no) |
|  |  | Last 12-month admissions | Admissions in the hospital over the last 12 months (number and yes/no) |
|  |  | Last 12-month primary care visits | Not applicable |

All variables in green were collected in the study. Variables on red were not selected due to its low applicability in the hospital setting.

Acronyms: ADR: adverse drug reaction; BMI: body mass index; DRP: drug related problem.

**REFERENCES:**

1) Carole P Kaufmann, Dominik Stämpfli, Kurt E Hersberger, Markus L Lampert. Determination of risk factors for drug-related problems: a multidisciplinary triangulation process. BMJ Open. 2015; 5(3): e006376. 2) Muaed Jamal Alomar. Factors affecting the development of adverse drug reactions (Review article). Saudi Pharm J. 2014 Apr; 22(2): 83–94. 3) Ferrández O, Grau S, Urbina O, Mojal S, Riu M, Salas E. Validation of a score to identify inpatients at risk of a drug-related problem during a 4-year period. Saudi Pharm J. 2018 Jul;26(5):703–8. 4) Mongaret C, Quillet P, Vo TH, Aubert L, Fourgeaud M, Michelet-Huot E, et al. Predictive factors for clinically significant pharmacist interventions at hospital admission. Medicine (Baltimore). 2018 Mar; 97(9): e9865. 5) Tomás Vecina S, García Sánchez L, Pascual Arce B, Riera Paredesl I. Pharmacist intervention program to improve patient safety in an emergency department. Emergencias. 2010;22(2):85-90.
