# Supplementary material for: Drug related problems in clinical practice: a cross-sectional study on their prevalence, risk factors and associated pharmaceutical interventions
Source: Sci Rep. 2021 Jan 13;11:883. doi: 10.1038/s41598-020-80560-2 (PMC7807048; doi:10.1038/s41598-020-80560-2)
Supplement: Supplementary file 3 — Supplementary Information 3. [file 41598_2020_80560_MOESM3_ESM.docx]

**Appendix 3. Distribution of the type of drug-related problems (DRP) across therapeutic groups (ATC: anatomical therapeutic chemical classification system)**

| **Type of DRP and ATC group** | **Number of DRP** | |  |
| --- | --- | --- | --- |
| **Adverse drug event (allergic)** | **19** | |  |
| Blood and blood forming organs | 1 | |  |
| Antiinfectives for systemic use | 7 | |  |
| Musculo-skeletal system | 3 | |  |
| Nervous system | 7 | |  |
| Various | 1 | |  |
| **Adverse drug event (non-allergic)** | **144** | |  |
| Alimentary tract and metabolism | 14 | |  |
| Blood and blood forming organs | 18 | |  |
| Cardiovascular system | 34 | |  |
| Genito-urinary system and sex hormones | 2 | |  |
| Systemic hormonal preparations, excluding sex hormones and insulins | 9 | |  |
| Antiinfectives for systemic use | 31 | |  |
| Antineoplastic and immunomodulating agents | 3 | |  |
| Nervous system | 27 | |  |
| Respiratory system | 5 | |  |
| Various | 1 | |  |
| **Drug treatment more costly than necessary** | **26** | |  |
| Blood and blood forming organs | 16 | |  |
| Antiinfectives for systemic use | 6 | |  |
| Antineoplastic and immunomodulating agents | 2 | |  |
| Nervous system | 2 | |  |
| **Effect of drug treatment not optimal** | **216** | |  |
| Alimentary tract and metabolism | 17 | |  |
| Blood and blood forming organs | 11 | |  |
| Cardiovascular system | 35 | |  |
| Genito-urinary system and sex hormones | 3 | |  |
| Systemic hormonal preparations, excluding sex hormones and insulins | 3 | |  |
| Antiinfectives for systemic use | 84 | |  |
| Antineoplastic and immunomodulating agents | 12 | |  |
| Musculo-skeletal system | 1 | |  |
| Nervous system | 39 | |  |
| Respiratory system | 3 | |  |
| Sensory organs | 5 | |  |
| Various | 3 | |  |
| **No effect of drug treatment/therapy failure** | **80** | |  |
| Alimentary tract and metabolism | 6 | |  |
| Blood and blood forming organs | 5 | |  |
| Cardiovascular system | 6 | |  |
| Systemic hormonal preparations, excluding sex hormones and insulins | 3 | |  |
| Antiinfectives for systemic use | 36 | |  |
| Antineoplastic and immunomodulating agents | 1 | |  |
| Musculo-skeletal system | 1 | |  |
| Nervous system | 17 | |  |
| Respiratory system | 2 | |  |
| Sensory organs | 1 | |  |
| Various | 2 | |  |
| **Toxic adverse drug-event** | **291** | |  |
| Alimentary tract and metabolism | 18 | |  |
| Blood and blood forming organs | 16 | |  |
| Cardiovascular system | 34 | |  |
| Systemic hormonal preparations, excluding sex hormones and insulins | 2 | |  |
| Antiinfectives for systemic use | 143 | |  |
| Antineoplastic and immunomodulating agents | 23 | |  |
| Musculo-skeletal system | 10 | |  |
| Nervous system | 38 | |  |
| Respiratory system | 4 | |  |
| Sensory organs | 1 | |  |
| Various | 2 | |  |
| **Unclear problem/complaint. Further clarification necessary** | **84** | |  |
| Alimentary tract and metabolism | 6 | |  |
| Blood and blood forming organs | 8 | |  |
| Cardiovascular system | 15 | |  |
| Systemic hormonal preparations, excluding sex hormones and insulins | 4 | |  |
| Antiinfectives for systemic use | 12 | |  |
| Antineoplastic and immunomodulating agents | 25 | |  |
| Musculo-skeletal system | 1 | |  |
| Nervous system | 10 | |  |
| Respiratory system | 2 | |  |
| Various | 1 | |  |
| **Unnecessary drug-treatment** | **29** | |  |
| Alimentary tract and metabolism | 3 | |  |
| Blood and blood forming organs | 2 | |  |
| Cardiovascular system | 4 | |  |
| Genito-urinary system and sex hormones | 2 | |  |
| Antiinfectives for systemic use | 6 | |  |
| Antineoplastic and immunomodulating agents | 2 | |  |
| Musculo-skeletal system | 2 | |  |
| Nervous system | 6 | |  |
| Respiratory system | 1 | |  |
| Sensory organs | 1 | |  |
| **Untreated indication** | **272** | |  |
| Alimentary tract and metabolism | 32 | |  |
| Blood and blood forming organs | 36 | |  |
| Cardiovascular system | 63 | |  |
| Dermatologicals | 1 | |  |
| Genito-urinary system and sex hormones | 23 | |  |
| Systemic hormonal preparations, excluding sex hormones and insulins | 2 | |  |
| Antiinfectives for systemic use | 7 | |  |
| Antineoplastic and immunomodulating agents | 7 | |  |
| Musculo-skeletal system | 9 | |  |
| Nervous system | 55 | |  |
| Antiparasitic products, insecticides and repellents | 2 | |  |
| Respiratory system | 5 | |  |
| Sensory organs | 28 | |  |
| Various | 2 | |  |
| **Wrong effect of drug treatment** | **20** | |  |
| Alimentary tract and metabolism | 3 | |  |
| Blood and blood forming organs | 1 | |  |
| Cardiovascular system | 4 | |  |
| Systemic hormonal preparations, excluding sex hormones and insulins | 2 | |  |
| Antiinfectives for systemic use | 5 | |  |
| Nervous system | 5 | |  |
| **Missing data** | | **4** | |
|  |  | |  |
| **Total** | **1185** | |  |

NOTE: There were 4 missing values regarding the specific drug in the database. There was one missing value in each of the following categories “toxic drug event”, “untreated indication, “effect of drug treatment not optimal” and “unnecessary drug treatment”.
